# *Pseudomonas aeruginosa* Can Be Detected in a Polymicrobial Competition Model Using Impedance Spectroscopy with a Novel Biosensor

**DOI:** 10.1371/journal.pone.0091732

**Published:** 2014-03-10

**Authors:** Andrew C. Ward, Patricia Connolly, Nicholas P. Tucker

**Affiliations:** 1 Department of Biomedical Engineering, University of Strathclyde, Glasgow, United Kingdom; 2 Strathclyde Institute of Pharmacy and Biomedical Sciences, University of Strathclyde, Glasgow, United Kingdom; Institut Pasteur, France

## Abstract

Electrochemical Impedance Spectroscopy (EIS) is a powerful technique that can be used to elicit information about an electrode interface. In this article, we highlight six principal processes by which the presence of microorganisms can affect impedance and show how one of these - the production of electroactive metabolites - changes the impedance signature of culture media containing *Pseudomonas aeruginosa.* EIS, was used in conjunction with a low cost screen printed carbon sensor to detect the presence of *P. aeruginosa* when grown in isolation or as part of a polymicrobial infection with *Staphylococcus aureus*. By comparing the electrode to a starting measurement, we were able to identify an impedance signature characteristic of *P. aeruginosa*. Furthermore, we are able to show that one of the changes in the impedance signature is due to pyocyanin and associated phenazine compounds. The findings of this study indicate that it might be possible to develop a low cost sensor for the detection of *P. aeruginosa* in important point of care diagnostic applications. In particular, we suggest that a development of the device described here could be used in a polymicrobial clinical sample such as sputum from a CF patient to detect *P. aeruginosa*.

## Introduction

Electrochemical Impedance Spectroscopy (EIS) has been investigated extensively as a tool for the detection of microbial attachment and biofilm formation [1]–[11]. However, few examples exist where this technology has been used in conjunction with a low cost screen printed sensor in a point of care device. One example is the use of EIS in conjunction with a low cost screen printed electrode to detect the level of moisture in chronic wounds, without the need to remove the dressing. This device minimises the need to disrupt the dressing unnecessarily therefore reducing patient discomfort and enhancing clinical practice decisions about dressing replacement [12]. These wound moisture sensors have also been investigated in the context of wound infection to determine if it is possible to detect different strains of *Staphylococcus aureus* growing in a suspension in Mueller-Hinton Broth with different concentrations of glucose [13]. In this study, it was found that specific impedance signatures could be discerned between control electrodes and those inoculated with *S. aureus* once growth increased above 5×10^7^ CFU/ml.

**Figure 1:**
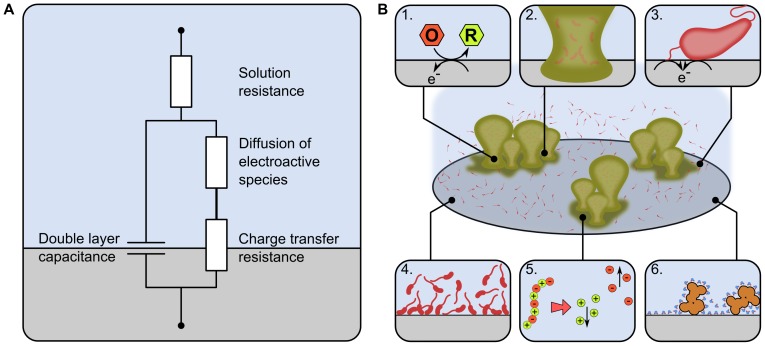
Mechanisms through which microorganisms could affect the impedance. (A) The impedance signature of a given electrochemical system is defined by the interplay of solution resistance, redox compounds, diffusion gradients and the electrolyte composition adjacent to the electrode surface. (B) Microorganisms could affect this impedance signature through: (1) production of electroactive secondary metabolites that facilitate a charge transfer at the electrode/electrolyte interface; (2) biofilm matrix attached to the electrode surface that affects capacitance and/or charge transfer; (3) direct microbial attachment, through pili, flagella and outer membrane proteins facilitating charge transfer; (4) Outer cell membrane contact at high cell densities that affect capacitance; (5) Breakdown of nutrients within the media reducing solution resistance; (6) Protein/macromolecule adsorption on the electrode surface influencing double layer capacitance.

Impedance spectroscopy measures the resistance and reactance of an electrode in contact with an electrolyte across a spectrum of AC frequencies. A number of electrochemical processes affect these two measurements at different frequencies and these can be interpreted by a simple model called the Randles equivalent circuit [14], [15] (figure 1A). The ability of the electrode to exchange electrons with the electrolyte is governed by the electrode material and the composition of the media. The availably of the electroactive species at the electrode surface has an impact on the electron transfer rate across the interface (and therefore the impedance) in a diffusion dependant manner. Other processes which affect impedance include the dielectric properties of the electrolyte adjacent to the electrode and the resistance of the bulk electrolyte. The former is known as the double layer capacitance and is defined by the relationship between polar and non-polar molecules and structures in very close proximity to the electrode. The latter is defined by the number of ions and other charged compounds within the media that convey charge between the electrodes. We propose six potential mechanisms through which the electrode-electrolyte interface could be changed by the growth of microorganisms, thus influencing impedance is a characteristic manner. These are: the production of redox compounds [16]–[19]; the deposition of biofilm material on the electrode surface; charge transfer through the attachment of cells and microbial nanowires [6], [20]–[23]; the presence of microbial cells in close proximity to the electrode surface; breakdown of nutrients within the electrolyte [24]–[26]; and the adsorption of proteins to the electrode surface [15], [27] (figure 1B).

The impedance spectrum can be interpreted by analysing the reactance and resistance data directly from the measurement or by analysing the modulus or phase angle of the impedance (see supporting information S1 for an explanation of how these are derived). Numerous circuit models have been proposed to explain the underlying processes that give rise to a particular impedance profile related to bacterial attachment and biofilm formation [1]–[3], [6], [8], [11], [28], [29]. In this study we used the normalisation procedure discussed by Farrow (2012), which allows relative changes in the impedance over time to be analysed by dividing a parameter of interest against its corresponding value at the start of the experiment [30]:
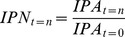



Where 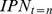 is the normalised impedance parameter of interest (i.e. reactance, resistance, impedance modulus or phase) and 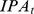 is the absolute (as measured) impedance parameter. The aim of the normalised impedance parameter is to identify a characteristic signature that can be used to detect a specific microorganism. An example is the clinically important pathogen *Pseudomonas aeruginosa*.

*P. aeruginosa* is an opportunistic pathogen capable of inhabiting numerous environments, including the cystic fibrosis (CF) lung, burns patients and babies in hospital neonatal units [31]–[34]. Early detection of *P. aeruginosa* within the CF airway may be useful by enabling prompt treatment and prevention of further lung damage. *P. aeruginosa* produces numerous virulence factors such as pyocyanin, which plays a role in pathogenicity and host tissue damage. The virulence of pyocyanin has been shown in several studies to be multi-faceted, including the inhibition of ciliary beating and cellular respiration, induction of neutrophil apoptosis and depletion of cellular glutathione [35]. In this study, we have investigated the application of EIS to a label-less, low cost screen printed sensor for the detection of *P. aeruginosa* and have elucidated at least one of the causes for the observed changes in impedance.

We used a novel sensor to determine if it is possible to detect the presence of *P. aeruginosa* in a simple polymicrobial model with *Staphylococcus aureus. S. aureus* was used because it is also commonly found within the CF airway [36]. The bacteria were cultured in an artificial sputum media (ASM) made with large quantities of mucin and DNA [37] designed to simulate conditions within the CF airway. EIS was used to sweep across a frequency spectrum from 100 mHz to 1 MHz with a custom built measurement chamber containing the electrode, which remained in-situ throughout the experiment.

## Results and Discussion

### Baseline Impedance Characteristics of the Electrodes show a High Initial Impedance Signature

Electrodes were designed with a working electrode that was much smaller than the counter electrode and constructed using thick film screen printing techniques with a carbon ink printed onto acetate. Carbon ink was used because of its low cost, which is an important driver for a point of care, disposable sensor. Silver chloride (Ag-AgCl) ink was also considered, however results from another study suggest that screen printed Ag-AgCl electrodes may affect bacterial growth [13]. The carbon nature of the electrodes resulted in very high magnitudes of impedance, with the impedance modulus ranging from 600 Ω at high frequency to 4.8 MΩ at 100 mHz. It was recognised that the high impedance might mask some of the impedance changes caused by the bacteria. This was traded off against the low cost of producing the sensors and the avoidance of Ag-AgCl ink in the electrode design. Therefore, in order to effectively analyse differences between inoculated and control samples, the normalisation procedure described above was used in conjunction with the phase angle and impedance modulus.

### *P. aeruginosa* is Specifically Detectable Alone, or as Part of Coculture with *S. aureus*

Impedance experiments were carried out to characterise the typical signatures resulting from microbial broths containing the *P. aeruginosa* strain PA14 and *S. aureus* strain RN4220, grown alone and together. Electrode chambers were inoculated in replicates of at least four with PA14, RN4220, a combination of the two, or left sterile as negative controls. For electrodes inoculated with *P. aeruginosa* or *S. aureus*, equal numbers of colony forming units (CFUs) were used for the starting density, between 3×10^6^ and 5×10^6^ CFU/ml. Measurements were taken periodically for a total of three days. After inoculation, the electrodes were placed in a microaerophilic environment, more representative of conditions within the lungs [37].

**Figure 2:**
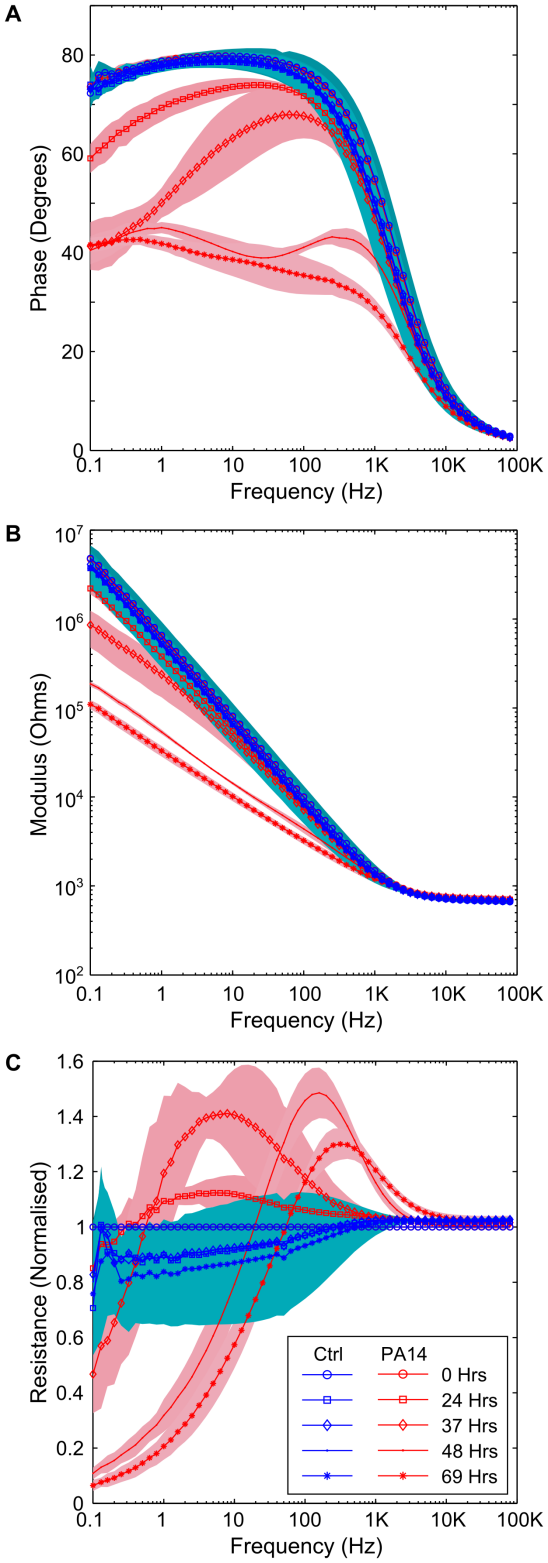
Changes in the impedance resulting from *P. aeruginosa* PA14. (A) phase plot, showing a drop in the absolute phase angle as a result of microbial growth, (B) the final impedance modulus at low frequency is more than one and a half orders of magnitude lower than the starting impedance, (C) the normalised resistance shows a peak for the electrode chambers containing PA14 that is not present in the control data. Background shading and error bars represent +/−1 SD. PA14 *n* = 4; Neg Ctrl *n* = 5.

At 24 hours, changes in the phase angle and impedance modulus were clearly observable between the samples inoculated with PA14 and control samples (figure 2). Similar behaviour has been observed with other *P. aeruginosa* strains across multiple experiments, including PAO1 grown in LB, and J1385 and J1532: a non-mucoid and mucoid isogenic pair from a CF patient [38] (data not shown). The impedance modulus drops from 4.7 MΩ to 100 KΩ over that course of the experiment at low frequencies (0.1 Hz), where charge transfer and mass diffusion of electroactive compounds are the most dominant impedance processes. A drop in the phase angle from around 90° in the electrodes containing PA14 cultures shows that the interface changes from one which has no electron transfer to one where a proportion of the current across the electrode-electrolyte interface is carried through electron transfer. Specifically, it can be seen that the growth of *P. aeruginosa* results in a current carried partly by the capacitance of the interface and partly by electron transfer at frequencies below 1000 Hz (after 24 hours). Corresponding changes are also observable in a complex plane plot, where an arc is evident at higher frequencies, indicative of the presence of a charge transfer resistance [14]. In contrast, the impedance signature of the negative controls remains constant throughout the experiment, with no deviation from a capacitive interface (i.e. an interface where no electron transfer occurs). Interestingly, a peak is visible in the normalised resistance data from the experiment (figure 2C). Peaks of this nature might be characteristic of *P. aeruginosa* and could be used as a powerful tool in a diagnostic device for the identification of different bacteria.

**Figure 3:**
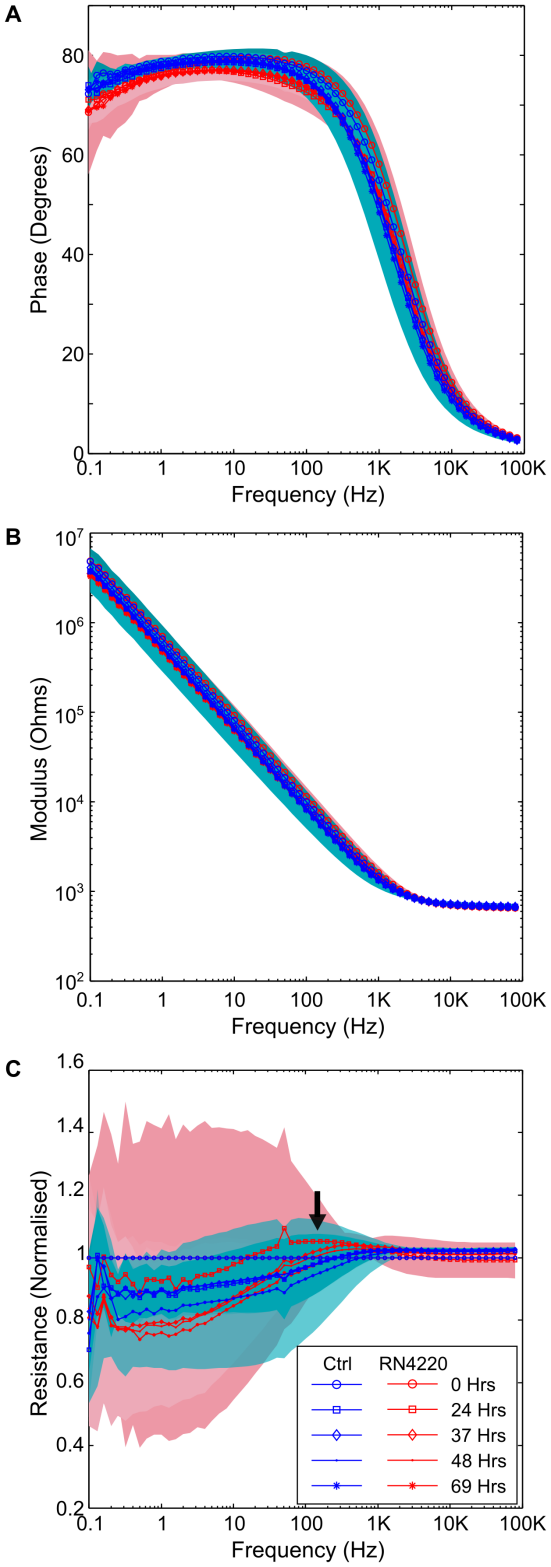
Impedance changes as consequence of growth with *S. aureus* RN4220. Shows that there is no discernible different in (A) the phase, (B) the modulus and (C) the normalised reactance under the same conditions as those used for *P. aeruginosa* PA14. The arrow indicates a slight peak at 125 Hz at 24 hours moving to 400 Hz at later timepoints. Background shading represent +/−1 SD. RN4220 *n* = 4; Neg Ctrl *n* = 5.

We decided to investigate the impact that the growth of *S. aureus* RN4220 would have upon the impedance signature, to determine if it would change in a similar manner to that seen with PA14. It was observed that the interface did not change significantly throughout the experiment, compared to the negative control, for either the raw impedance data or any of the normalised impedance data (figure 3). In order to test this further, *S. aureus* was grown in Tryptone Soya Broth (TSB) for 3 days in aerobic conditions. These growth conditions resulted in a cell density of 5.58×10^7^ CFU/ml at the end of the experiment compared to a starting cell density of 1.47×10^7^ CFU/ml. It was noted in a subsequent experiment that the cell density of *S. aureus* in a culture was higher (i.e. 4.56×10^8^ CFU/ml) if the media was vigorously disrupted with a 1 ml pipette prior to taking an aliquot for colony counting. As with the experiments using ASM, no change was found in the impedance when RN4220 was grown in TSB (data not shown).

Previously, investigators identified that changes in the impedance were a result of the breakdown of nutrients within the media [24]–[26], in a field that became known as impedance microbiology. When *S. aureus* was grown in ASM or TSB our sensor did not result in a significant change in impedance. This is probably because the impedance microbiology methods measure changes in the conductivity of the media, an effect not seen here due to the large baseline impedance. The large baseline impedance would also screen any small changes to the electrode-broth interface impedance that were caused by the bacteria. Although not significantly different, the normalised resistance plot shows that the samples containing *S. aureus* do differ in their impedance curve, with a slight peak visible after 37 hours at 400 Hz (figure 3C). An electrode with lower starting impedance might be more sensitive to these changes and enable a unique signature to be identified for *S. aureus*.

**Figure 4:**
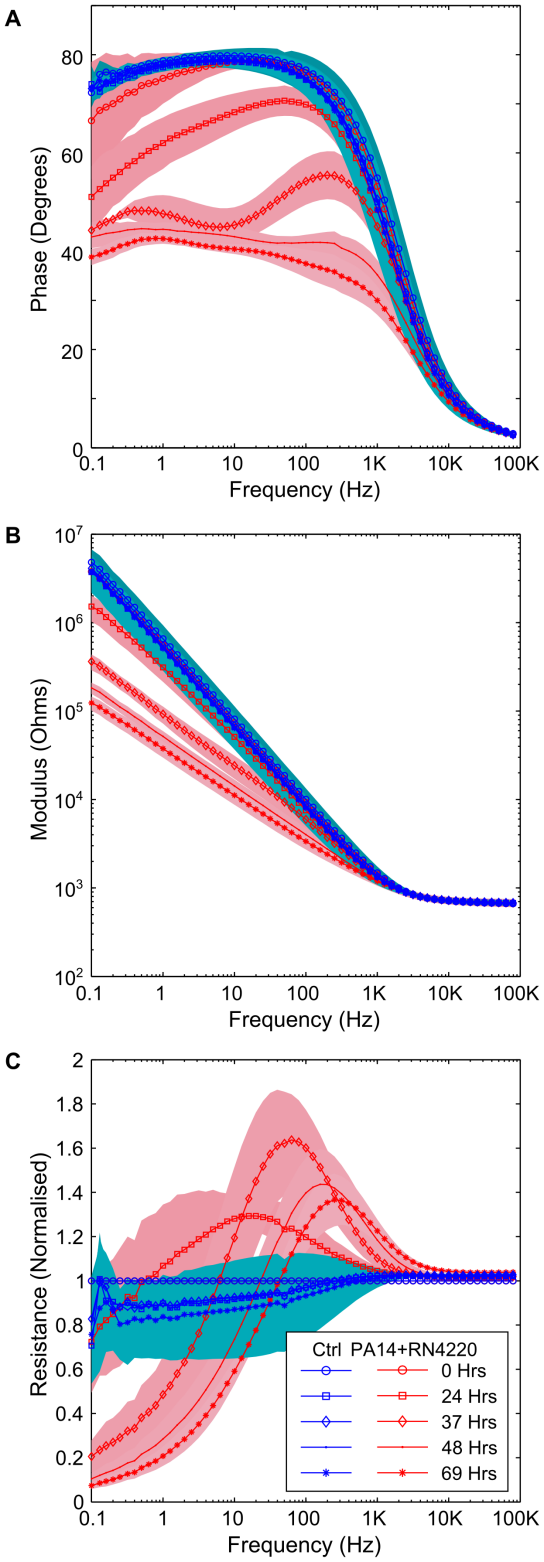
Polymicrobial growth of *P. aeruginosa* and *S. aureus*. When RN4220 and PA14 are grown together, the impedance is similar to when PA14 is grown alone. (A) and (B) a change is clearly visible the overall phase angle and a drop in the impedance modulus, (C) the normalised resistance shows a peak, and a change in the peak frequency as a result of growth. Background shading represent +/−1 SD. Polymicrobial *n = *5; neg ctrl *n* = 5.

A more representative situation within the CF airway is a polymicrobial infection consisting of two or more microbial species [36]. In order to test the performance of the sensor in this context, measurements were taken with PA14 and RN4220 grown together at equal starting densities in the same electrode chamber, under microaerophilic conditions. As with the monoculture of *P. aeruginosa* alone, changes in the impedance between the negative control and the polymicrobial infection were observable after 24 hours in the impedance phase and modulus data (figure 4A and 4B). These changes were similar to those observed with PA14 alone and by the end of the experiment, the polymicrobial infection model and PA14 had a similar impedance curve. The normalised resistance data was also similar, suggesting that PA14 was responsible for the changes seen in the impedance (figure 4C).

To further understand the dynamics of the polymicrobial model, colony counting was carried out on an aliquot of the samples taken at 37 hours and 69 hours (table 1), which indicates the number of viable planktonic cells present. It can be seen that PA14 outcompetes RN4220 by the end of the experiment, at which point no RN4220 could be cultured from the electrode chambers. This supports other work that found *P. aeruginosa* results in reduced numbers of gram positive bacteria in a polymicrobial infection model [39]. At the 37 hour timepoint, it can also be seen that fewer viable RN4220 cells exist within the polymicrobial samples. In contrast, at 37 hours 3.65×10^5^ CFU/ml were present for RN4220, indicating that some viable cells remained in the presence of PA14, but no growth occurred.

**Table 1:** Indicative cell densities of planktonic cells at the end of the experiment and timepoints during the experiment.

	Cell Concentration (CFU/ml)		
Sample Type	Initial (0 hrs)	37 Hrs mean (range)	69 Hrs mean (range)
PA14 Mono	3–5×10^6^	6.5×10^8^ (5.4×10^8^–7.3×10^8^)	2.1×10^8^ (6.5×10^7^–3.3×10^8^)
RN4220 Mono	3–5×10^6^	1.6×10^7^ (1.1×10^7^–2.9×10^7^)	3.1×10^7^ (4.9×10^6^–7.2×10^7^)
PA14 Poly	3–5×10^6^	6.0×10^8^ (4.1×10^8^–7.2×10^8^)	8.5×10^7^ (5.9×10^6^–1.0×10^8^)
RN4220 Poly	3–5×10^6^	3.7×10^5^ (6.8×10^4^–1.4×10^6^)	0

### Pyocyanin is Present in the Supernatant and Causes Changes in Impedance

It is well documented that a number of electroactive phenazine pigments are produced by *P. aeruginosa* [16], [40], [41]. One of these phenazines, pyocyanin has been studied extensively in several fields, from its effects as a virulence factor in a clinical context [35] to its use as a redox mediator in a glucose sensor [42]. We hypothesised that the green pigment observed in the media at the end of a 69 hours experiment was partly caused by the blue colour from the redox active phenazine, pyocyanin. To explore this further, after 69 hours the media was removed from the electrode chambers and filter sterilised. The supernatant was then added to an equal volume of methanol and the UV-visible spectrum was measured.

**Figure 5:**
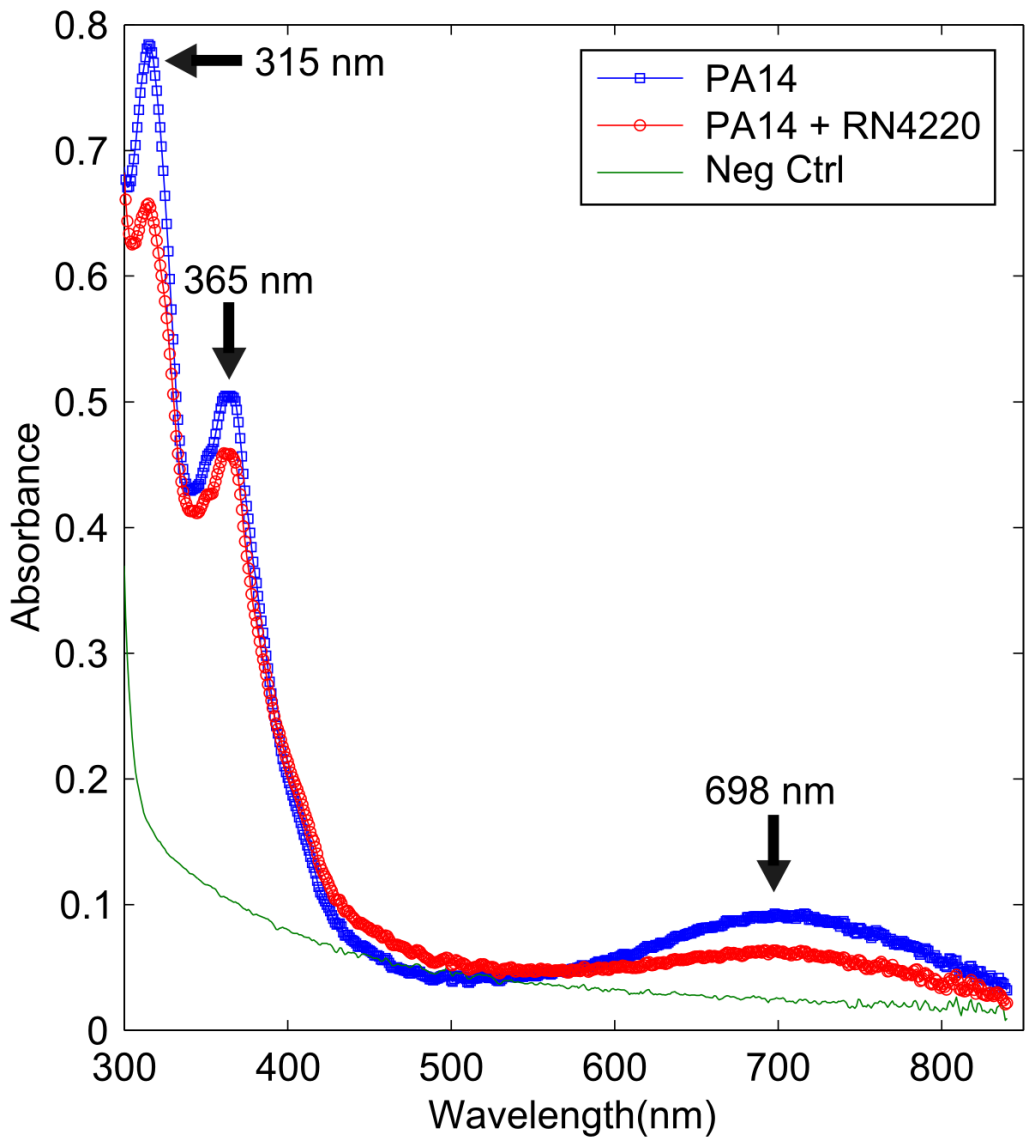
UV-visible spectroscopy of the supernatant after 3 days showing typical spectrum. At the end of experiments, the supernatant was taken from all samples and combined with 50% MeOH. The peaks highlighted by the arrows are similar to those found in pyocyanin, a redox active phenazine produced by *P. aeruginosa*.

Peaks were observed in the UV-visible spectrum for media where PA14 was grown, either alone or as part of the polymicrobial infection model (figure 5). These changes were observed at 315 nm, 368 nm and 698 nm, and correspond to the absorbance peaks of pyocyanin found at 238 nm, 316 nm, 347 nm, 368 nm and 690 nm [43]. It is clear therefore from the UV-visible spectrum that pyocyanin is present within the ASM media following the growth of *P. aeruginosa* at 69 hours. We investigated the impact that pyocyanin had on the measured impedance by firstly measuring the impedance of pyocyanin alone in ASM at different concentrations, from 1000 µM to 10 µM. Only a slight change in the impedance was seen at low frequencies and high concentrations of pyocyanin (1000 µM to approximately 500 µM), which was unrepresentative of the changes observed when the impedance of a culture containing PA14 (alone or polymicrobial) was measured (data not shown). We reasoned that this could be because the pyocyanin existed in its oxidised form and therefore no reduced pyocyanin was available to balance the redox reaction at each electrode. Alternatively, pyocyanin could have been just one of multiple electroactive compounds that transferred electrons in a chain, with the compounds at an appropriate redox potential compared to the electrode causing the change in impedance. An alternative situation was explored, were pyocyanin was added directly to a late log culture of *P. aeruginosa* and *S. aureus* in order to assess the effect it had on the impedance.

**Figure 6:**
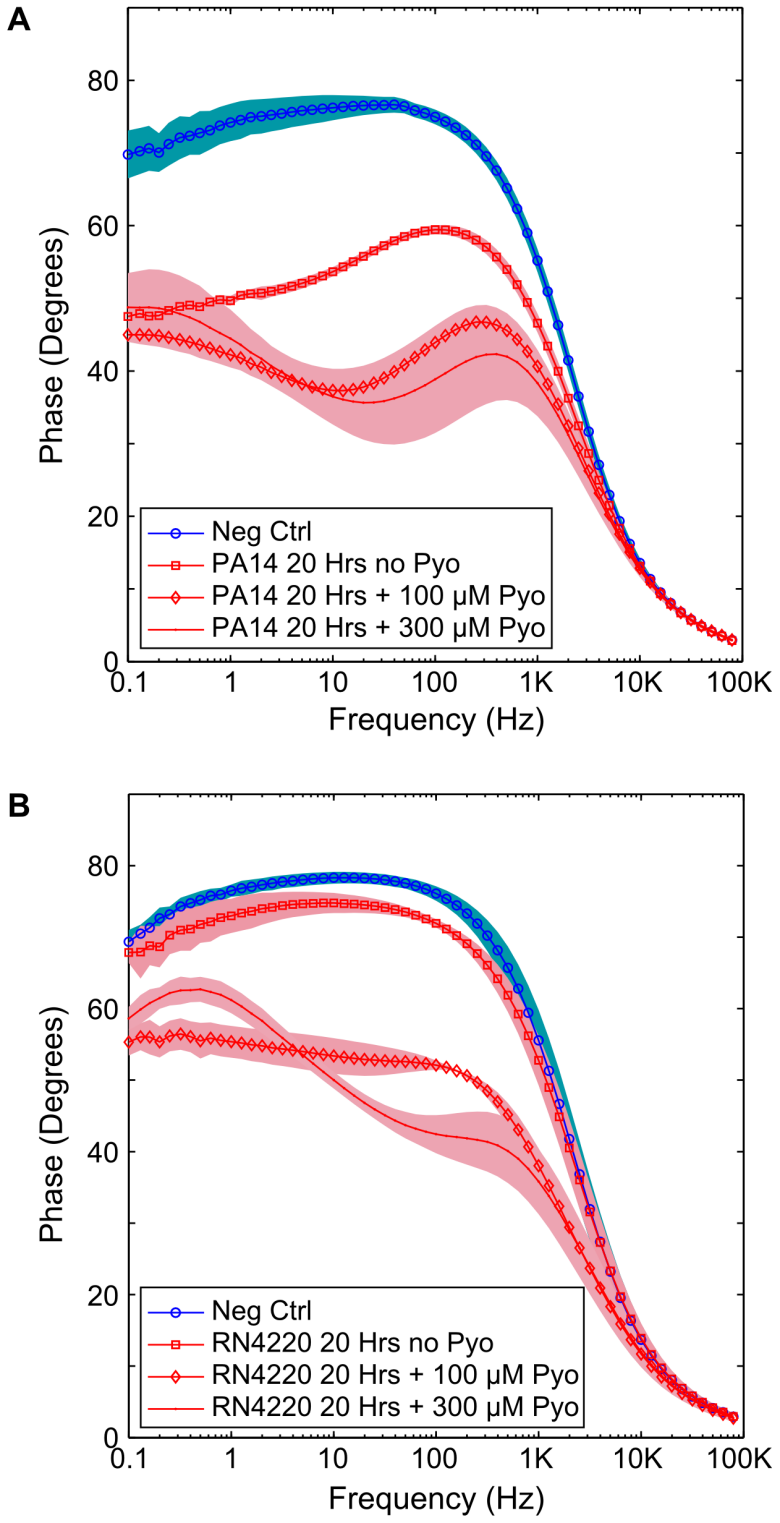
Addition of Pyocyanin yields a distinct change in the impedance. Adding the phenazine pigment pyocyanin to cultures of *P. aeruginosa* and *S. aureus* causes the impedance to change. (A) Change in impedance of PA14, which is similar to the change caused by growth in microaerophilic conditions. (B) Change in impedance of *S. aureus*, RN4220. Background shading represents +/−1 SD, *n* = 3.

Cultures of *P. aeruginosa* (PA14) and *S. aureus* (RN4220) were grown in ASM microaerophilically overnight to late log phase in 24 well plates. After measuring the impedance of the media, and the impedance of the late log phase cultures, pyocyanin was added to the cultures at concentrations of 100 µM and 300 µM and a further impedance measurement was immediately carried out. The results indicated a clear change in the impedance for each of the electrode chambers (figure 6), similar to the changes in phase observed through the growth of PA14 and the polymicrobial infection model. It was noticed when similar experiments were carried out in LB media that the blue pigment resulting from the addition of pyocyanin changed from blue to clear in all but the top layer of each of the cultures. On shaking, the pyocyanin was rapidly oxidised and returned to its original blue pigment, before gradually being reduced again. This was apparent after five minutes for the culture of *S. aureus* while *P. aeruginosa* took longer to lose its pigment and did so to a lesser degree.

These results indicate that pyocyanin plays a role in the observed impedance of PA14 when grown microaerophilically in ASM. Furthermore, it is clear that the presence of *S. aureus* leads to the reduction of pyocyanin in some manner and a consequent change in the impedance signature. It has been shown previously that pyocyanin is capable of non-enzymatically oxidising NADH in a 2*e* ^−^, 2H^+^ reaction [44], and has more recently been explored in terms of the ability of phenazines to facilitate extracellular respiration through electron shuttling [40]. The reduction of pyocyanin by these mechanisms could explain how the microorganisms have the capacity to change the impedance.

**Figure 7:**
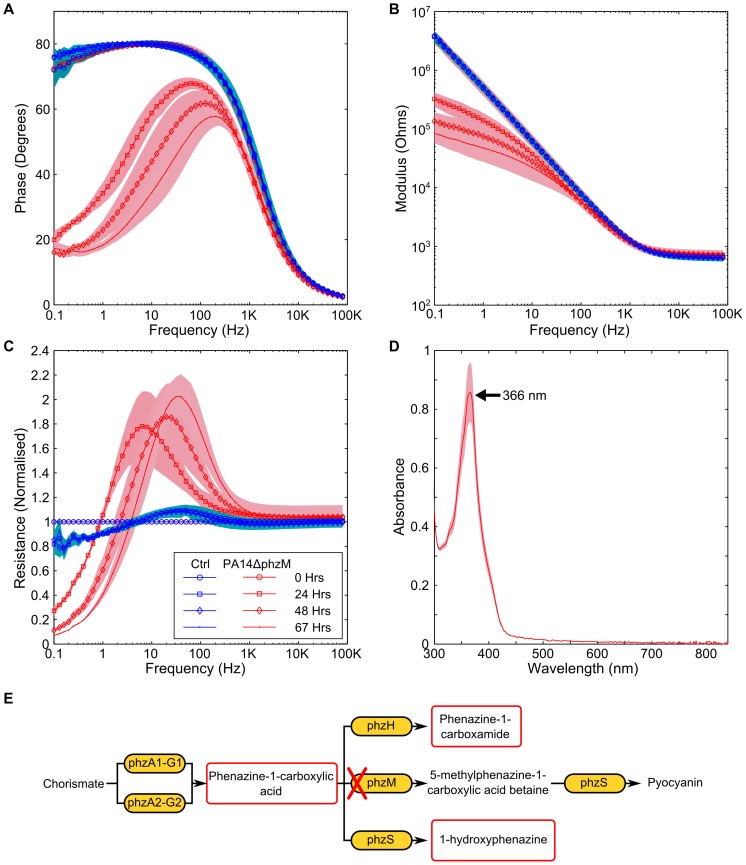
Impedance changes caused by the PA14ΔphzM mutant. Changes to the impedance spectrum can be clearly observed when a mutant of *P. aeruginosa*, unable to produce pyocyanin is measured. Changes to the (A) phase, (B) modulus and (C) normalised resistance are similar to those found in the PA14 wild type. (D) the UV-visible spectrum shows a peak at 367 nm, suggesting the presence of phenazine-1-carboxylic acid. (E) Indicates the biosynthesis pathway for phenazines and shows the effect that the phzM knockout is anticipated to have. Background shading represent +/−1 SD. PA14ΔphzM *n* = 3; Neg Ctrl *n* = 3.

**Figure 8:**
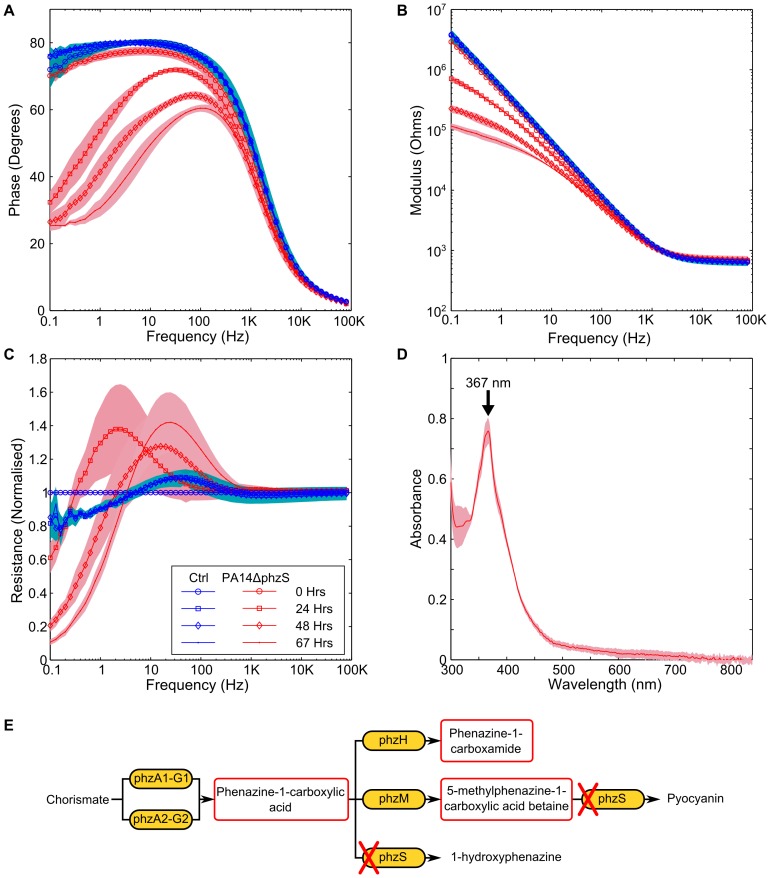
Impedance changes caused by the PA14ΔphzS mutant. Similar changes are observable in the (A) phase, (B) modulus and (C) normalised resistance to the wild type and the PA14ΔphzS mutant. (D) the presence of phenazine-1-carboxylic acid can also be seen. (E) Indicates the biosynthesis pathway for phenazines and shows the effect that the phzS knockout is anticipated to have. Background shading represent +/−1 SD. PA14ΔphzS *n* = 3; Neg Ctrl *n* = 3.

**Figure 9:**
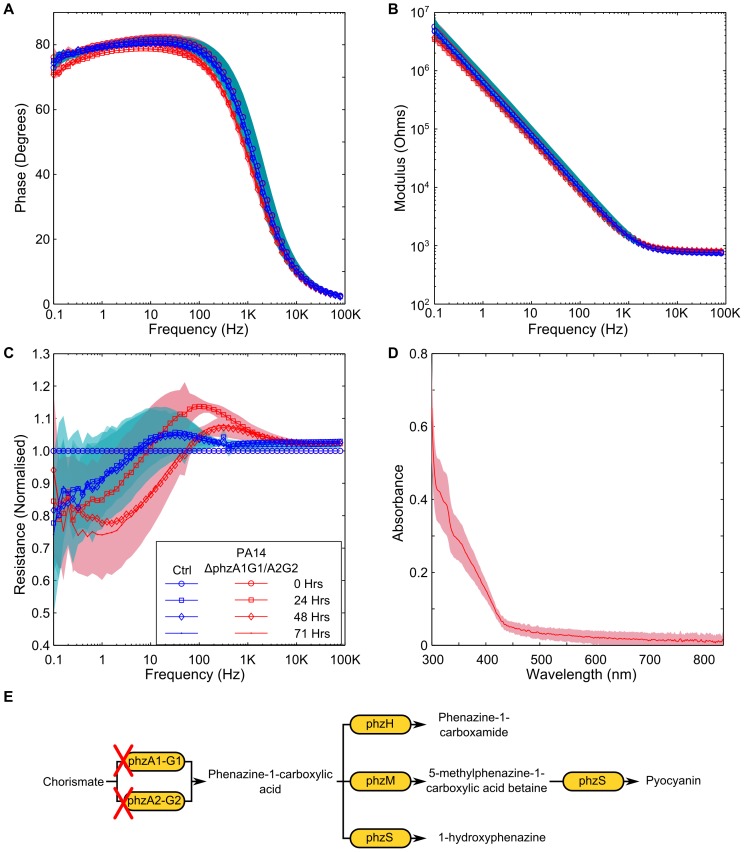
Measurement of P. aeruginosa PA14ΔphzA1-G1/A2-G2 double mutant. (A and B) no significant different can be seen in the phase or modulus of the impedance. (C) a small difference in the normalised resistance can be seen, contrasted to the negative control. (D) The UV-visible spectrum indicates that the phenazines present in the wild type or other mutants tested are no present. (E) Indicates the point on the biosynthesis pathway that the double knockout is anticipated to affect. Background shading represent +/−1 SD. PA14ΔphzA1-G1/A2-G2 *n* = 4; Neg Ctrl *n = *4.

The synthesis pathway of pyocyanin in *P. aeruginosa* is also responsible for the production of phenazine-1-carboxylic acid, 1-hydroxyphenazine, phenazine-1-carboxamide and 5-methylphenazine-1-carboxylic acid betaine [41], [45]–[47]. Like pyocyanin, these phenazines are redox active [16] and therefore could also have an impact upon the impedance signature. In order to explore the potential changes that a culture of *P. aeruginosa* unable to produce pyocyanin would have on the impedance, we explored the impedance signature of mutant strains of PA14 from the PA14NR set [48] and also the impedance of a double knockout mutant [47]. PA14ΔphzM a mutant that produces phenazine-1-carboxylic acid and 1-hydroxyphenazine and PA14ΔphzS is a mutant that produces phenazine-1-carboxylic acid and 5-methylphenazine-1-carboxylic acid betaine, were tested [46]. Whilst both strains were able to produce phenazine, neither of the strains were able to produce pyocyanin. In order to explore the impedance of *P. aeruginosa* when no phenazines were produced, we tested a strain where the phzA1-G1 and phzA2-G2 operons had been deleted [47]. A change in the impedance phase angle and modulus, and a peak in the normalised resistance were observed in data for both the PA14ΔphzM and PA14ΔphzS strains tested (figures 7A–C and 8A–C). The UV-visible spectra shows a peak clearly visible at 366 nm, suggesting the presence of phenazine-1-carboxylic acid [46]. No peak is present at 698 nm, confirming that no pyocyanin was produced (figure 7D and 8D). Although the PA14ΔphzM and PA14ΔphzS strains followed the same basic pattern as the wild type strain, a different impedance signature can clearly be seen, particularly in the phase angle and the normalised resistance. This shows that different combinations of phenazine compounds influence the electrode/electrolyte interface (figure 7E and 8E). In contrast, the PA14ΔphzA1-G1/A2-G2 mutant did not result in a significant impedance change in the phase and modulus data (figure 9A and B). A small peak in the normalised resistance data, different to the negative control, is clearly visible after 24 hours, although this is not of the same magnitude as the wild type or other mutants tested. This suggests a process other than electroactive transfer mediated by phenazines. Further investigation is required to explore the underlying mechanism leading to this peak.

### Microbial Attachment to the Electrode Surface may also have an Impact on Impedance

**Figure 10:**
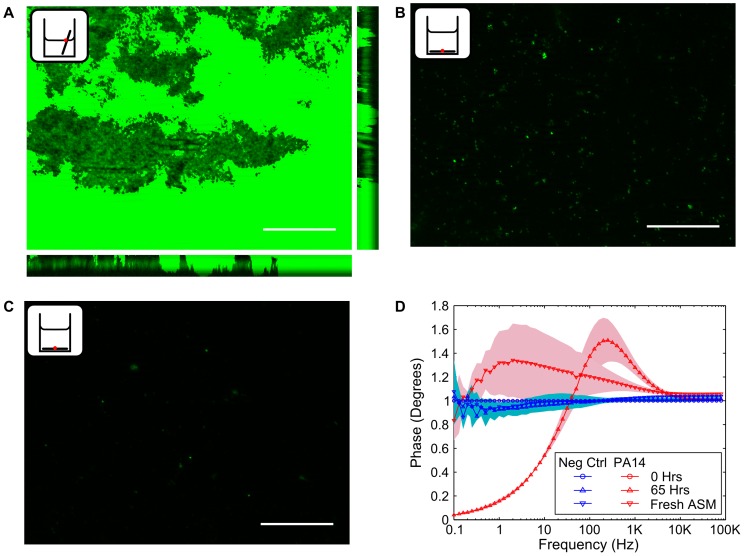
Attachment of *P. aeruginosa* PA14 to the electrode surface could affect the impedance. Electrode coupons were incubated in LB media (aerobic conditions) for 48 Hrs. (A) At the air-liquid interface, extensive biofilm formation can be seen indicating that *P. aeruginosa* will attach to the electrode surface. (B) Some limited microbial attachment can be seen on the electrode surface at the bottom of the a well after 48 hours. (C) Negative control showing that the electrode surface does not autofluoresce. (D) A small change can be observed in the impedance signature (contrasted to the negative control) at the end of a microaerophilic experiment when the media is exchanged with a fresh, sterile aliquot. Scale bars represent 50 um. Shading in (D) represents +/−1SD, *n = *3.

Epifluorescent microscopy of the electrode surface was used in order to determine the extent to which *P. aeruginosa* is capable of attaching to the electrode surface. Coupons of the carbon electrode material were incubated aerobically for 48 hours in LB media. It can be seen that at the air-liquid interface, extensive biofilm formation on the carbon electrode surface is possible (figure 10A). This shows that *P. aeruginosa* has the capacity to attach to the electrode surface and form a biofilm. In contrast, incubating the electrode coupon at the bottom of a chamber resulted in some microbial attachment, but little biofilm formation (figure 10B). This could have been due to the viscous, thick media at the end of the experiment pulling biofilm away from the electrode surface when the cell was aspirated. The negative control indicates that there is no autofluorescence on the electrode surface (figure 10C).

We investigated the impedance signature of *P. aeruginosa* at the end of a 3 day microaerophilic incubation period in ASM, when the media within the electrode chamber was replaced with fresh media. Under these conditions, we noticed that the media at the end of the experiment was far less viscous and the biofilm formed at the air liquid interface was much more limited. Interestingly, we found that a small change in impedance could still be distinguished against control samples (figure 10D). This could suggest that microbial attachment to the electrode surface also plays a role in the observed impedance changes. However, it is interesting to note that the normalised resistance peak observed with PA14ΔphzA1-G1/A2-G2 (figure 9C) is different to the normalised resistance observed when the media is replaced (figure 10D). This could be related to the residual media and limited biofilm material attached to the electrode containing electroactive compounds produced by *P. aeruginosa* changing the impedance in addition to the effect observed with the PA14ΔphzA1-G1/A2-G2 mutant.

### Conclusion

This article describes how *P. aeruginosa* could be detected using a low cost, disposable sensor. The technique can be applied with low cost sensors and microelectronic measurement equipment, making it potentially useful in different point of care diagnostic scenarios, such as monitoring of CF sputum. However, several challenges still need to be overcome in the translation of this technology from the laboratory to a point of care application. For example, in the case of CF, sputum samples are thick and viscous, requiring some form of homogenisation prior to measurement and a change in the redox state of the electroactive phenazines between expectoration and measurement might affect the sensitivity of the sensor. Future investigations will therefore focus upon device testing in real clinical samples in order to identify and address potential detection problems.

## Materials and Methods

### Media, Buffers and Microbial Strains

LB media (used to grow overnight cultures, for the adjustment of optical density at the start of each experiment and for epifluorescent microscopy) was made by mixing 1 g tryptone (order code: BPE1421, Fisher Scientific) 0.5 g yeast extract (order code: 92144, Fluka) and 0.5 g NaCl (order code: 31434, Sigma-Aldrich) with 100 ml of dH_2_O before autoclaving for 20 mins at 121°C**.** TSB (order code: CM0129, Fisher Scientific) was used as directed and autoclaved prior to use. 100 ml aliquots of ASM were produced following an adapted version of the protocol by Kirchner et al. (2012). Briefly, 400 mg DNA from fish sperm (order code: 74782, Sigma-Aldrich) and 500 mg of porcine mucin (order code: M2378, Sigma-Aldrich) were sterilised overnight in 2 ml of 100% EtOH. This was then poured into 50 ml of sterile dH_2_O. 25 mg of the specified amino acids, 1 ml egg yolk emulsion (order code: 17148, Sigma-Aldrich) and 500 mg NaCl was then added to the DNA and porcine mucin as described [37]. The pH of the media was tested and adjusted to 6.9 by aseptically taking small aliquots of media to measure the pH after adjusting with sterile 1 M Tris. Normal saline was used for electrode conditioning and was made in aliquots of 100 ml by mixing dH_2_O with 0.9 g NaCl. The strains used in this experiment are described in table 2. Mutants taken from a copy of the PA14NR transposon library [48] and were cultured for single colonies on LB agar plates containing 15 µg/ml of gentamician.

**Table 2:** Bacterial strains used in this study.

Strain	Comment	Reference
RN4220	*S. aureus* wild type laboratory strain	[51]
PA14WT	*P. aeruginosa* wild type laboratory strain	[48]
PA14ΔphzS	*P. aeruginosa* PA14 mutant from the PA14NR set (gene ID: 44099)	[48]
PA14ΔphzM	*P. aeruginosa* PA14 mutant from the PA14NR set (gene ID: 40343)	[48]
PA14ΔphzA1-G1/A2-G2	*P. aeruginosa* double knockout mutant	[47]

#### Electrode printing and preconditioning

The electrode pattern consisted of a small working electrode (2 mm diameter) surrounded by a larger counter electrode (10 mm diameter). Electrodes were manufactured using thick film screen printing processes on a DEK 247 semi-automatic screen printing machine (DEK). This was achieved firstly by producing a mask for the electrode pattern. The electrodes were then screen printed onto acetate sheets suitable for a mono laser printer (order code: 9543383, Supplies Team). Electrodes were printed using Electrodag PF407A carbon ink and were cured (after each printing cycle) in a box oven at 120°C for 30 minutes. A dielectric ink was hand painted over the track leading up to the working electrode with Gwent D2020823D2 and cured at 80°C for 30 minutes. After production, a 20 mm section of 15 ml centrifuge tube (order code: 11542293, Fisher Scientific) was mounted over the top of the electrode with Servisol silicone adhesive (order code: RE89W, Maplin), completing the electrode assembly (figure S1). An electrode conditioning procedure described by Wang et al. (1996) [49] was adapted as follows. 2 ml 0.9% NaCl made with dH_2_O was added to each electrode. Conditioning was carried out using a Solartron 1260 impedance gain/phase analyser and the Zplot/Zview software package. Using a 2 mm diameter platinum counter electrode (order code: PT007920, Goodfellows), both the working and counter carbon electrodes were DC biased at 2 volts and held there for 3 minutes, and then at −2 volts for a further 3 minutes. The electrode assemblies were sterilised by submerging in 70% EtOH for 10 minutes. Following this, the EtOH was allowed to fully evaporate in a sterile environment overnight.

#### Microaerophilic chamber design

A microaerophilic environment was created by using a lock ‘n’ lock plastic box (product number: HPL826M) modified with two 9 way D-sub connectors mounted through the sidewalls of the box (Order code: 674-0760 and 450-9258, RS components) to enable impedance measurements to be carried out without the need to remove the lid. The D-sub connectors were made airtight by sealing around the connectors on the inside of the box with Servisol silicone adhesive.

#### Calculation of starting cell densities and electrode inoculation

The number of PA14 and RN4220 CFUs at OD_600_ were determined through the production of a standard curve in a preparatory experiment. Overnight cultures of PA14 and RN4220 were grown in LB media and adjusted to an OD_600_ of 0.33 and 0.67 respectively, representing a cell density of approximately 3×10^8^ to 5×10^8^ CFU/ml. 1 ml of ASM media was then inoculated with 10 µl of the OD adjusted media.

#### Monoculture and polymicrobial experiments

At the start of the experiment, nine sterile electrodes were connected, inoculated and placed into each box, with up to 18 electrodes used in one experiment. At least four replicates of each sample (including negative control) were carried out. A microaerophilic environment was created using a Campygen gas generation pack (order code: 10108012, Fisher Scientific). Electrodes were then incubated at 37°C in a shaking incubator at 75 rpm for a maximum of three days.

#### Impedance measurement

All impedance measurements were carried out on a Solartron 1260 impedance gain/phase analyser in conjunction with the Zplot/Zview software package (Scribner Associates). Impedance measurements were carried out at the start of the experiment and then at least once every 24 hours for the duration of the experiment. The microaerophilic chamber was removed from the incubator and kept at room temperature for the duration of the measurements which typically lasted approximately 45 minutes per chamber and four minutes per electrode. The impedance measurements carried out in the experiment were made using a Solartron 1260 impedance analyser with an AC rms voltage of 200 mV between 0.1 Hz and 1 MHz. Impedance data analysis was carried out using Matlab r2012b (Mathworks).

#### UV-Vis spectrum and associated protocol

At the end of the experiment period, the supernatant was filter sterilised through 0.22 µm syringe filter (Millipore) and then and added to an equal volume of methanol to fix the sample. The UV visible spectrum was then measured from 190 nm to 850 nm using a Thermofisher 2000x spectrophotometer. Data analysis of the UV-visible spectrum was carried out in Matlab r2012b (Mathworks).

#### Exogenous pyocyanin measurements

Pyocyanin purified from *P. aeruginosa* (order code: P0046, Sigma-Aldrich) was solubilised in 100% EtOH to create a 10x stock solution. This was then mixed with sterile dH_2_O for the measurements carried out using *S. aureus* and *P. aeruginosa*. 1 ml samples of *P. aeruginosa* and *S. aureus* (as monocultures) were grown overnight (20 hours) along with negative controls in 24 well plates. Following this, the samples were removed from the microaerophilic growth chamber and after initial impedance measurements, pyocyanin was added to aliquots of media to a final concentration of 100 µM and 300 µM. An impedance measurement was carried out at each concentration.

#### Epifluorescent microscopy of electrode coupons

Electrode coupons were manufactured in the same manner as the electrodes used in the experiment. The coupons were made by cutting electrodes into a shape compatible with incubation in a 24 well plate. After sterilising in 70% EtOH for 10 minutes, 1 ml of LB was added to each sample. For the positive control electrodes, the samples were positioned at an angle so that a portion of the coupon was out of the media, thus facilitating biofilm growth at the air liquid interface. Samples were then inoculated with PA14 or left sterile as controls and incubated aerobically at 37°C, shaking at 75 rpm for 48 hours. Coupons were removed from the 24 well plate, aspirated and then stained with 5 µM of SYTO 9 (order code: S-34854, Life Technologies). Microscopy was carried out on a Zeiss Axio Imager Z1, with an exposure time of 750 ms per image. Brightness and contrast were made consistent across images and scales bars were added in Fiji image [50].

## Supporting Information

Figure S1 **Example of an assembled electrode chamber.**
(TIF)

Supporting Information S1 **Background on Electrochemical Impedance Spectroscopy.**
(DOC)
