# Supplementary figures and images for: Pseudomonas aeruginosa Can Be Detected in a Polymicrobial Competition Model Using Impedance Spectroscopy with a Novel Biosensor
Source: PLoS One. 2014 Mar 10;9(3):e91732. doi: 10.1371/journal.pone.0091732 (PMC3948879; doi:10.1371/journal.pone.0091732)

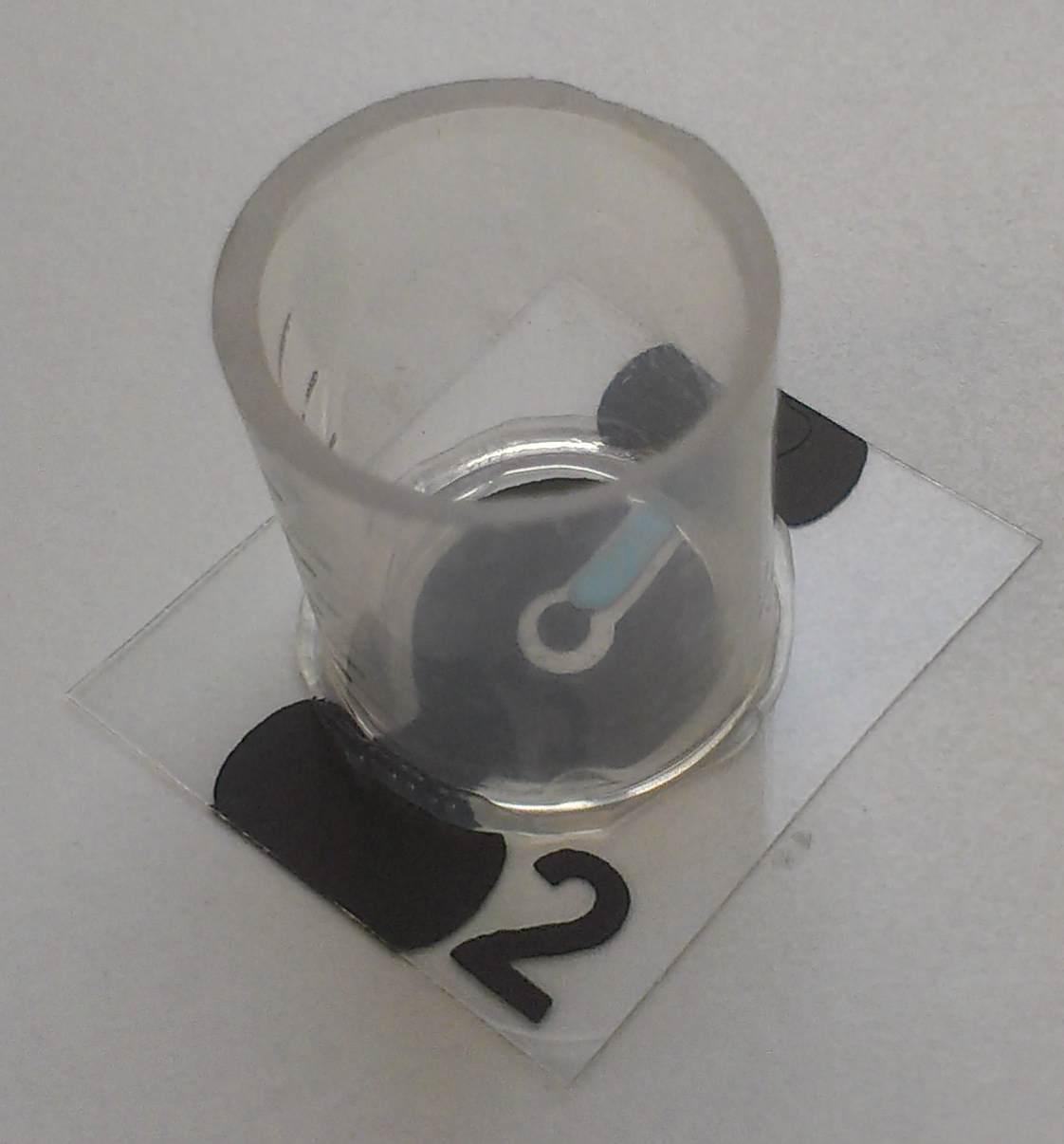

Supplement: Figure S1 — Example of an assembled electrode chamber. (TIF) [file pone.0091732.s001.tif]
