# Supplementary material for: Pseudomonas aeruginosa Can Be Detected in a Polymicrobial Competition Model Using Impedance Spectroscopy with a Novel Biosensor
Source: PLoS One. 2014 Mar 10;9(3):e91732. doi: 10.1371/journal.pone.0091732 (PMC3948879; doi:10.1371/journal.pone.0091732)
Supplement: Supporting Information S1 — Background on Electrochemical Impedance Spectroscopy. (DOC) [file pone.0091732.s002.doc]

***Pseudomonas aeruginosa* can be detected in a polymicrobial competition model using impedance spectroscopy with a novel biosensor**

**Andrew C Ward^1^, Patricia Connolly^1*^ and Nicholas P Tucker^2^**

^1^Department of Biomedical Engineering, University of Strathclyde, Glasgow, UK

^2^Strathclyde Institute of Pharmacy and Biomedical Sciences, University of Strathclyde, Glasgow, UK

*Corresponding author. Email: patricia.connolly@strath.ac.uk

**Supporting Information S1:** **Background on Electrochemical Impedance Spectroscopy**

**1. Background on Electrochemical Impedance Spectroscopy**

The term impedance is used to describe the obstruction to current presented by the whole system (i.e. combined contributions from resistance and reactance). Impedance can be usefully interpreted through the application of complex numbers where the resistance is treated as the real part and the reactance as the imaginary part, leading to:

|  | $Z=Z'-jZ''$ | [1] |
| --- | --- | --- |

Where$Z$is the overall impedance, $Z'$is the real (resistive) component,$Z''$is the imaginary (reactive) component and$j=\sqrt{-1}$. This complex number is related to the phase shift that occurs between the current and voltage in AC circuits with reactive components and can be shown in a complex (or Nyquist) plot. The complex number may also be expressed in polar form as follows:

|  | $Z=\left\vert Z\right\vert\angle\theta$ | [2] |
| --- | --- | --- |

Where$\left|Z\right|$is the modulus defined as the vector between the reactance and the resistance:

|  |  | [3] |
| --- | --- | --- |

Andis the phase angle, defined by:

|  |  | [4] |
| --- | --- | --- |

For a further background on the fundametals of resistance, capacitance and impedance, the reader is directed to Hughes and Smith (1995). For background on electrochemistry and impedance spectroscopy, Bard and Faulkner (2001) is recommended.

**2. References**

1. Hughes E, Smith IM (1995) Hughes electrical technology. Harlow [England]; New York, NY: Longman Scientific & Technical ; Wiley.

2. Bard AJ, Faulkner LR (2001) Electrochemical methods : fundamentals and applications. 2 edn. New York: Wiley.
